# Supplementary material for: Decreased liver B vitamin-related enzymes as a metabolic hallmark of cancer cachexia
Source: Nat Commun. 2023 Oct 6;14:6246. doi: 10.1038/s41467-023-41952-w (PMC10558488; doi:10.1038/s41467-023-41952-w)
Supplement: Supplementary file 1 — Supplementary Information [file 41467_2023_41952_MOESM1_ESM.pdf]

Supplementary Information

Decreased liver B vitamin-related enzymes as a metabolic hallmark of cancer cachexia

Yasushi Kojima[1], Emi Mishiro-Sato[1], Teruaki Fujishita[1], Kiyotoshi Satoh[2], Rie Kajino-Sakamoto[1], Isao Oze[3], Kazuki Nozawa[4], Yukiya Narita[4], Takatsugu Ogata[4], Keitaro Matsuo[3], Kei Muro[4], Makoto Mark Taketo[5], Tomoyoshi Soga[2], Masahiro Aoki[1, 6]

[1]Division of Pathophysiology, Aichi Cancer Center Research Institute, 1-1 Kanokoden, Chikusa-ku, Nagoya, Aichi 464-8681, Japan.
[2]Institute for Advanced Biosciences, Keio University, 246-2 Mizukami, Kakuganji, Tsuruoka, Yamagata 997-0052, Japan.
[3]Division of Cancer Epidemiology and Prevention, Aichi Cancer Center Research Institute, 1-1 Kanokoden, Chikusa-ku, Nagoya, Aichi 464-8681, Japan.
[4]Department of Clinical Oncology, Aichi Cancer Center Hospital, 1-1 Kanokoden, Chikusa-ku, Nagoya, Aichi 464-8681, Japan.
[5]Colon Cancer Project, Kyoto University Hospital-iACT, Kyoto University, Yoshida-Konoe-cho, Sakyo-ku, Kyoto 606-8501, Japan.
[6]Department of Cancer Physiology, Nagoya University Graduate School of Medicine, 65 Tsurumai-cho, Showa-ku, Nagoya, Aichi 466-8550, Japan

Correspondence: Yasushi Kojima, Division of Pathophysiology, Aichi Cancer Center Research Institute, 1-1 Kanokoden, Chikusa-ku, Nagoya, Aichi 464-8681, Japan. Phone: +81-52-762-6111; Fax: +81-52-763-5233; E-mail: ykojima@aichi-cc.jp

Correspondence: Masahiro Aoki, Division of Pathophysiology, Aichi Cancer Center Research Institute, 1-1 Kanokoden, Chikusa-ku, Nagoya, Aichi 464-8681, Japan. Phone: +81-52-762-6111; Fax: +81-52-763-5233; E-mail: msaoki@aichi-cc.jp

Fig. S1

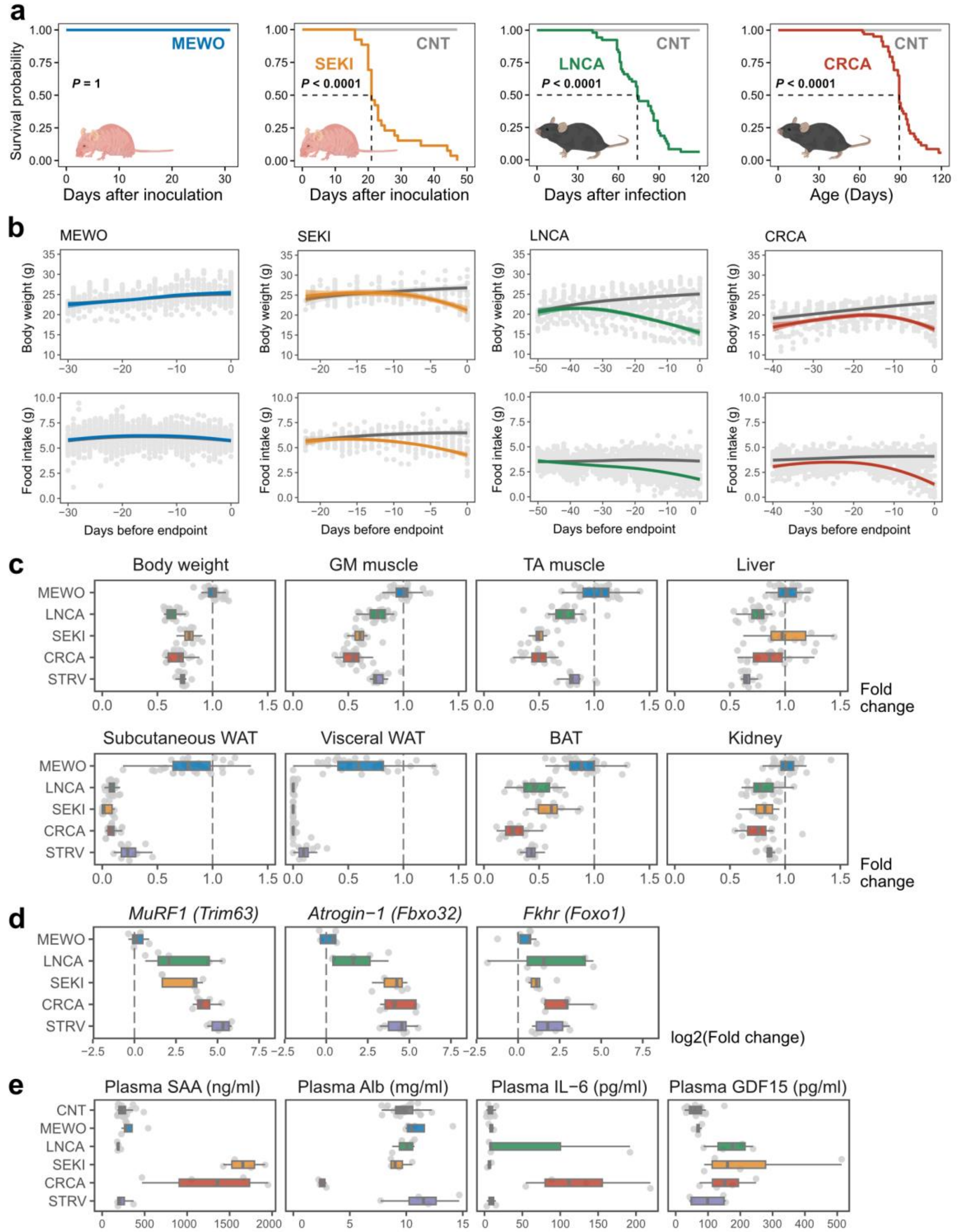

Fig. S1: SEKI, LNCA, and CRCA mice develop cancer cachexia. a, Kaplan-Meier survival curves for pooled mouse datasets from different experiments. Survival periods for MEWO, LNCA, and CRCA were capped at 30, 120, and 120 days, respectively. MEWO (N = 46 mice) with control mice (N = 32 mice); SEKI (N = 26 mice, MST (median survival time) = 21 days after inoculation) with control nude mice (N = 25 mice). LNCA

(N = 54 mice, MST = 74 days after infection) with control mice (CNT, N = 58 mice); CRCA (N = 72 mice, MST = 89 days old) with control mice (N = 97 mice). *P* values (Log rank test); MEWO, 1; LNCA, 3×10^{-13}; SEKI, 8×10^{-9}; CRCA, 2×10^{-16}. **b,** Changes in body weight and daily food intake. MEWO (N = 26, blue LOESS line) with control nude mice (N = 26 mice, grey LOESS line); SEKI (N = 9 mice, orange LOESS line) with control nude mice (N = 9 mice, grey LOESS line); LNCA (N = 16 mice, green LOESS line) with littermate controls (N = 16 mice, grey LOESS line); CRCA (N = 13 mice, red LOESS line, female only) with littermate controls (N = 13 mice, grey LOESS line, female only). LOESS, locally weighted scatterplot smoothing. **c,** Change in body weights and weights of gastrocnemius muscle (GA muscle), anterior tibialis muscle (TA muscle), liver, subcutaneous white adipose tissue (WAT), visceral WAT, brown adipose tissue (BAT), and kidney. MEWO, N = 31 mice; LNCA, N = 22 mice; SEKI, N = 12 mice; CRCA, N = 16 mice; STRV, N = 12 mice. **d,** Expression of *MuRF1*, *Atrogin-1*, and *Fkhr* mRNA in gastrocnemius muscle tissue analyzed by TaqMan PCR assay. MEWO, N = 5 mice; LNCA, N = 5 mice; SEKI, N = 5 mice; CRCA, N = 5 mice; STRV, N = 6 mice. **e,** Plasma levels of serum amyloid A (SAA), albumin (Alb), IL-6, and GDF15, measured by ELISA (enzyme-linked immunosorbent assay). MEWO, N = 4 mice; LNCA, N = 4 mice; SEKI, N = 4 mice; CRCA, N = 4 mice; STRV, N = 4 mice; CNT, pooled controls, N = 20 mice (MEWO control, N = 4 mice; LNCA control, N = 4 mice; SEKI control, N = 4 mice; CRCA control, N = 4 mice; STRV control, N = 4 mice). Boxplot style of FigS1, the middle line (median), the upper hinge (Q3, the third quantile), the lower hinge (Q1, the first quantile), the upper whisker (1.5 × (Q3 – Q1)), and the lower whisker (1.5 × (Q3 – Q1)). Source data are provided as a Source Data file.

Fig. S2

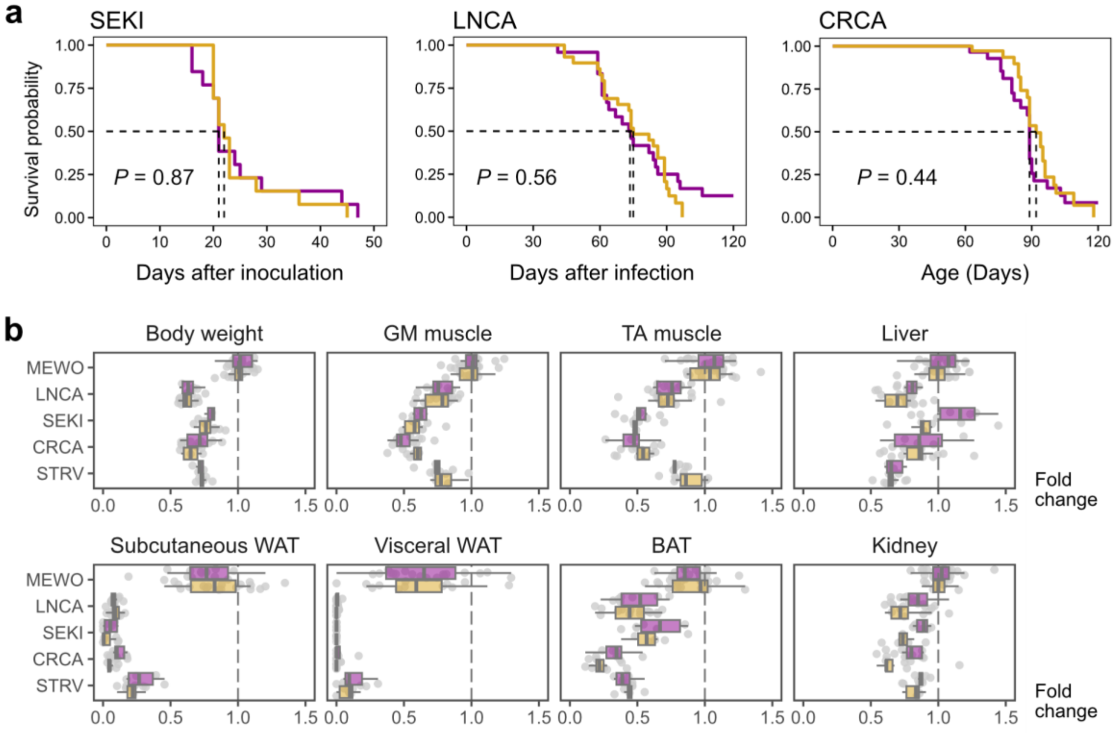

Fig. S2: LNCA, SEKI, and CRCA show no significant sex differences in survival or body compositional changes. a, Sex-based analysis of Kaplan-Meier survival curves for pooled mouse datasets from different experiments. Survival periods for LNCA and CRCA were capped at 120 days. Purple curve, female; Goldenrod curve, male. Female SEKI, N = 13 mice, MST (median survival time) = 21 days after inoculation; male SEKI, N = 13 mice, MST = 22 days after inoculation; female LNCA, N = 24 mice, MST = 73.5 days after infection; male LNCA, N = 30 mice, MST = 75 days after infection; female CRCA, N = 34 mice, MST = 85 days old; male CRCA, N = 38 mice, MST = 89 days old. Three *P*-values from the Log rank test. Note that Fig. S2a is a simple, sex-based analysis of the Fig. S1a dataset. **b,** Sex-based analysis of change in masses of body, gastrocnemius muscle (GM muscle), tibialis anterior muscle (TA muscle), liver, subcutaneous white adipose tissue (WAT), visceral WAT, brown adipose tissue (BAT), and kidney. Purple box, female; Goldenrod box, male. Female MEWO, N = 17 mice; male MEWO, N = 14 mice; female LNCA, N = 11 mice; male LNCA, N = 11 mice; female SEKI, N = 6 mice; male SEKI, N = 6 mice; female CRCA, N = 10 mice; male CRCA, N = 6 mice; female STRV, N = 6 mice; male STRV, N = 6 mice. Boxplot style, the middle line (median), the upper hinge (Q3, the third quantile), the lower hinge (Q1, the first quantile), the upper whisker (1.5 × (Q3 – Q1)), the lower whisker (1.5 × (Q3 – Q1)). Note that Fig. S2b is a simple, sex-based presentation of Fig. S1c. Source data are provided as a Source Data file.

Fig. S3

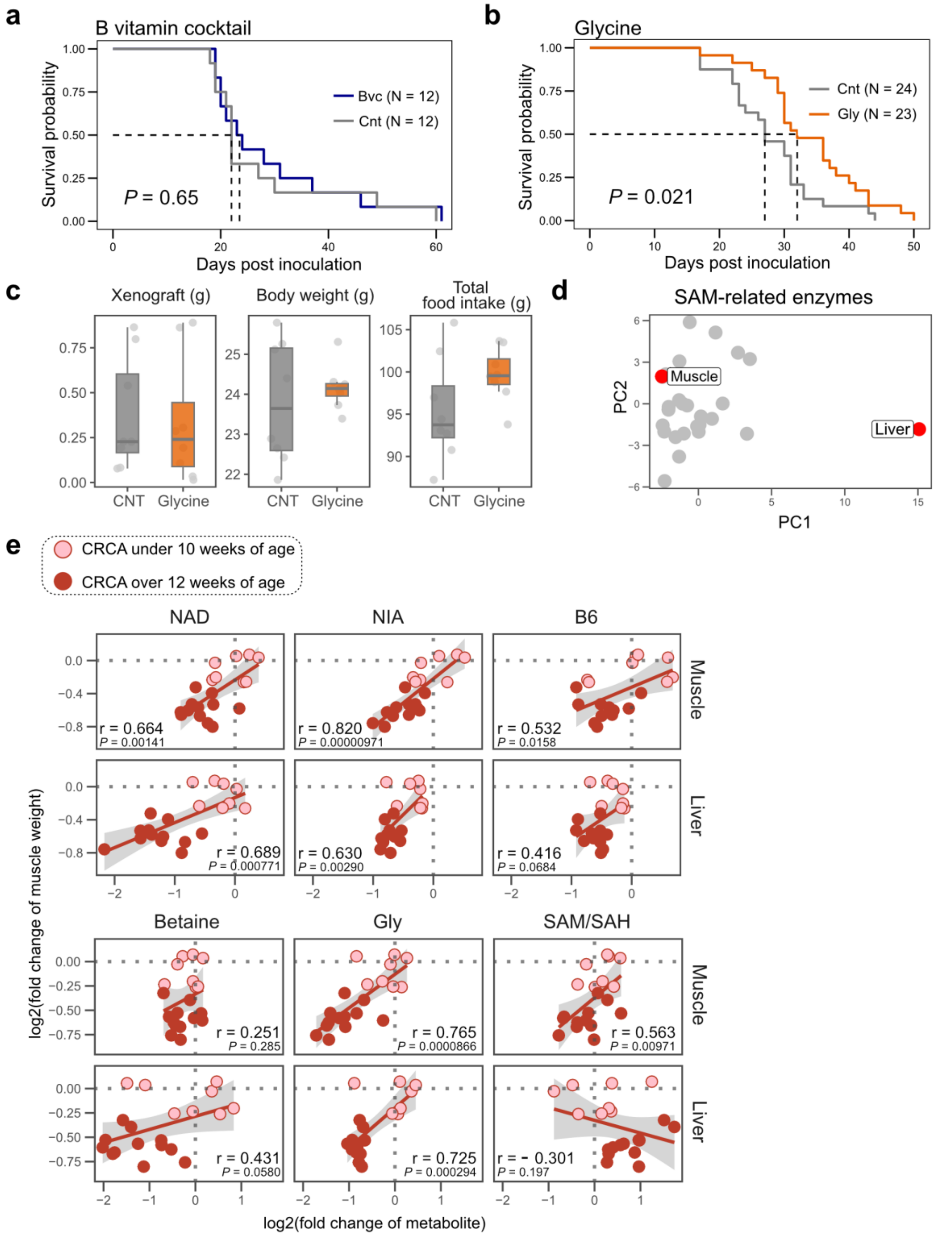

Fig. S3: CRCA model shows linear relationships between changes in body weight and changes in metabolite levels (NAD, NIA, B6, betaine, glycine, and SAM/SAH ratio). a, Kaplan-Meier survival curves of control SEKI mice (Cnt, N = 12 mice) and SEKI mice treated with a B vitamin cocktail (Bvc, N = 12 mice) based on a single experiment. Treatment with the B vitamin cocktail was started six days after inoculation of SEKI cells.

Log rank test, $P = 0.65$. Median survival time: control group, 22 days; B vitamin cocktail group, 23.5 days. **b,** Kaplan-Meier survival curves of control (Cnt, N = 24 female mice) and glycine-treated SEKI mice (Gly, N = 23 female mice) based on combined data from two independent experiments. Glycine administration was started five days after inoculation of SEKI cells. Log rank test, $P = 0.021$. Median survival time: control, 27 days; glycine, 32 days. The first experiment was significant (Cnt, N = 8 female mice; Gly, N = 8 female mice; Log rank test, $P = 0.023$), but the second experiment was not significant (Cnt, N = 16 female mice; Gly, N = 15 female mice; Log rank test, $P = 0.29$). **c,** Boxplots of xenograft, body weight, and total food intake. Data from SEKI experimental group sampled 17 days after starting of glycine administration. Control SEKI mice, N = 8 female mice; glycine-treated SEKI mice, N = 8 female mice. Total food intake, total food intake during glycine administration period (total 17 days). Welch's 2-sided *t*-test; xenograft, $P = 0.8152$; body weight, $P = 0.5378$; total food intake, $P = 0.111$. Boxplot style, the middle line (median), the upper hinge (Q3, the third quantile), the lower hinge (Q1, the first quantile), the upper whisker (1.5 × (Q3 – Q1)), the lower whisker (1.5 × (Q3 – Q1)). **d,** PCA plot of published mouse tissue/organ proteome data[1]. Tissues, N = 28 tissues; SAM enzymes, N = 46 enzymes. **e,** Scatterplots of changes in metabolite levels and gastrocnemius muscle weights in CRCA mice under 10 weeks of age (N = 8 female mice) and over 12 weeks of age (N = 12 female mice). C57BL/6N controls were used as references to calculate the fold change values (under 10 weeks of age, N = 7 female mice; over 12 weeks of age, N = 12 female mice). Note that the over 12 weeks of age group is a part of the CRCA group presented in Fig 1d. Red lines are produced by locally weighted scatterplot smoothing (LOESS). Gray zones, 95% confidence intervals. Horizontal and vertical dotted lines, log2(fold change) = 0. r, Pearson`s correlation coefficient; *P, P* value (Pearson's correlation). NAD, nicotinamide adenine dinucleotide; NIA, niacin, B6, vitamin B6; Gly, glycine; SAM, S-adenosyl methionine; SAH, S-adenosyl homocysteine; SAM/SAH, SAM/SAH ratio. Source data are provided as a Source Data file.

Fig. S4

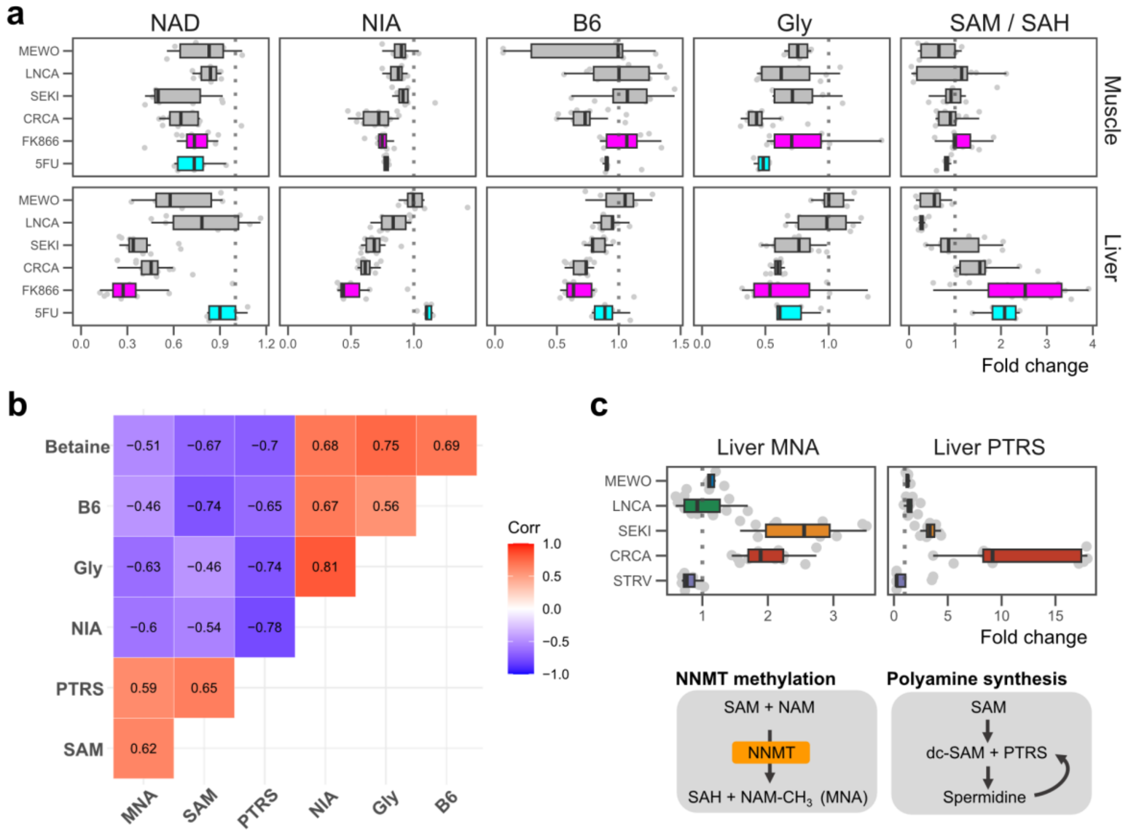

Fig. S4: Hepatic C1 metabolism is closely linked to two B vitamin subgroups (niacin and vitamin B6). a, Fold changes in NAD, niacin (NIA), vitamin B6 (B6), glycine (Gly), and SAM/SAH ratio with respect to control mice groups. FK866, NAMPT inhibitor; 5FU, fluorouracil. MEWO, N = 6 mice; LNCA, N = 11 mice; CRCA, N = 13 mice; 5FU, N = 5 mice; FK866, N = 11 mice; Muscle SEKI, N = 8 mice; Liver SEKI, N = 11 mice. Note that gray boxplot data are based on the same data used in Fig. 2a, b, e, and f. **b,** Pearson's correlation matrices generated from logarithmic combined liver metabolome data of the four experimental groups (MEWO, N = 6 mice; LNCA, N = 11 mice; SEKI, N = 11 mice; CRCA, N = 13 mice). Gly, glycine; NIA, niacin, B6, vitamin B6; MNA, 1-methylnicotinamide; PTRS, putrescine. **c,** Fold changes in 1-methylnicotinamide (MNA) and putrescine (PTRS). MEWO, N = 6 mice; LNCA, N = 11 mice; SEKI, N = 11 mice; CRCA, N = 13 mice; STRV, N = 8 mice. Schematic of NNMT methylation and polyamine synthesis pathways. NAM, nicotinamide; NNMT, nicotinamide N-methyltransferase; dc-SAM, decarboxylated SAM. Boxplot style, the middle line (median), the upper hinge (Q3, the third quantile), the lower hinge (Q1, the first quantile), the upper whisker (1.5 × (Q3 – Q1)), the lower whisker (1.5 × (Q3 – Q1)). Source data are provided as a Source Data file.

Fig. S5

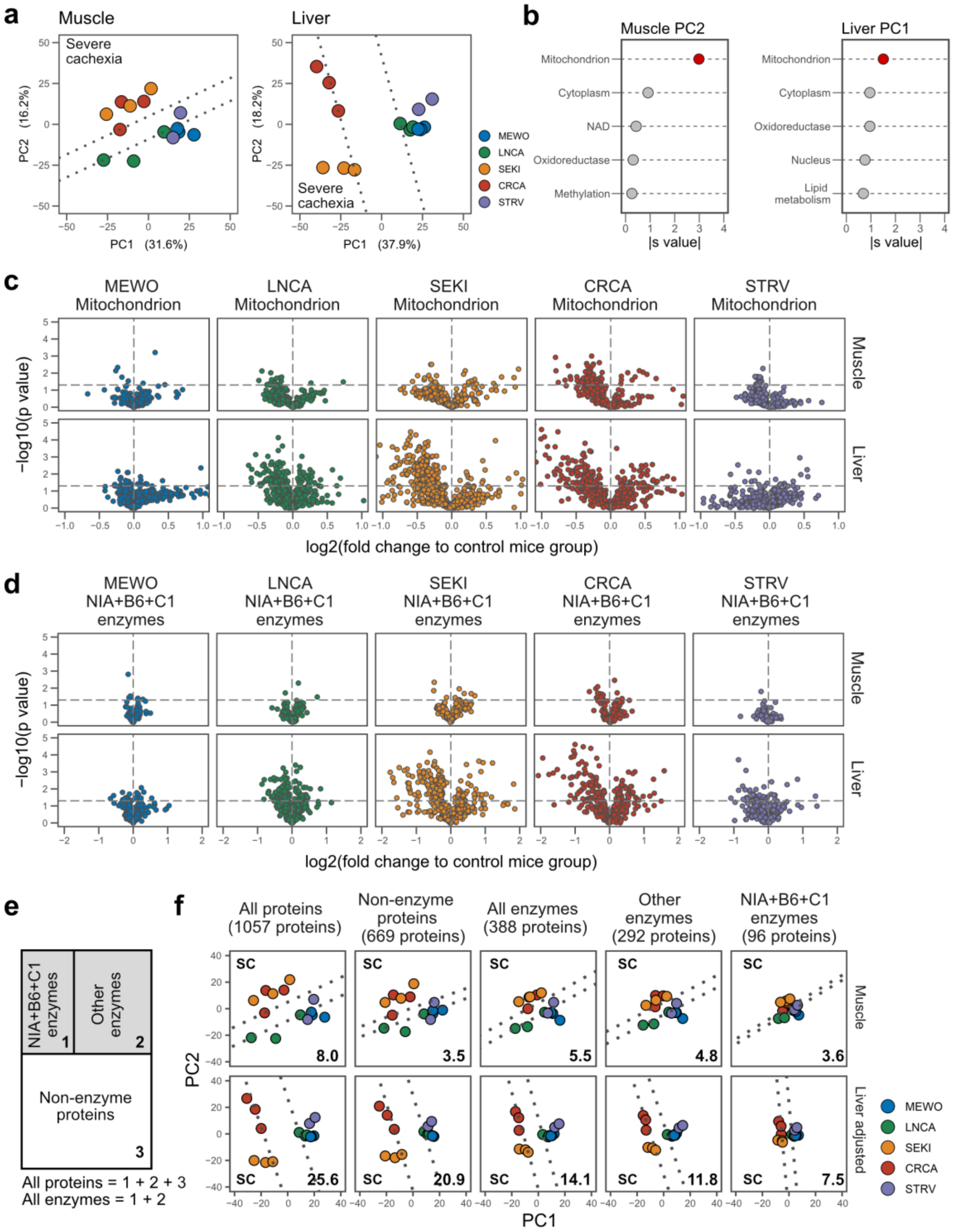

Fig. S5: Proteomic analysis reveals more drastic proteomic changes in liver than in muscle. a, PCA plots of muscle and liver proteomic data. Contribution ratios (%) of the first (PC1) and second principal components (PC2) are indicated in the axis titles, respectively. Dashed lines represent margins between the SEKI/CRCA group and the MEWO/LNCA group produced by linear SVMs. MEWO, N = 3 mice; LNCA, N = 3 mice;

SEKI, N = 3 mice; CRCA, N = 3 mice; STRV, N = 2 mice. **b,** Enrichment analysis of muscle PC2 and liver PC1 loadings using UniProt annotation. Abs. s value, absolute s value, a combination of *P*-value and effect size. Red, mitochondrion. Details of this enrichment analysis are described in the Methods section. PC loading data are also provided in the Source Data file for Fig. S5b. **c,** Keyword-based volcano plots of muscle and liver proteomic data (Table S5). Mitochondrion, proteins annotated with mitochondrion in UniProt database. Horizontal dashed lines show where $P = 0.05$ (Welch's 2-sided *t*-test). Plots are limited to a range from -1 to 1 on the X-axis and 0 to 5 on the Y-axis. MEWO, N = 3 mice; LNCA, N = 3 mice; SEKI, N = 3 mice; CRCA, N = 3 mice; STRV, N = 2 mice. **d,** Keyword-based volcano plots of muscle and liver proteomic data (Table S5). NIA+B6+C1 enzymes, NIA-, B6-, and C1-related enzymes. Horizontal dashed lines show where $P = 0.05$ (Welch's 2-sided *t*-test). Plots are limited to a range of -2 to 2 on the X-axis and 0 to 5 on the Y-axis. MEWO, N = 3 mice; LNCA, N = 3 mice; SEKI, N = 3 mice; CRCA, N = 3 mice; STRV, N = 2 mice. **e,** Schematic of three mutually exclusive compartments based on the UniProt annotations (Table S5). NIA+B6+C1 enzymes, NIA-, B6-, and C1-related enzymes. **f,** PCA plot of non-adjusted muscle and adjusted liver proteome datasets split by the UniProt annotations. Facet subtitles by Fig. S5e. Dashed lines represent margins between the SEKI/CRCA and MEWO/LNCA groups produced by linear SVMs. SC, SEKI and CRCA group area. The number inside the facet is the maximum margin, that is, the shortest Euclidean distance between the two margins on the plots. Note that the liver PCA plots are representative, and maximum margin values for liver data are the median from 1000 random samplings for adjustment. MEWO, N = 3 mice; LNCA, N = 3 mice; SEKI, N = 3 mice; CRCA, N = 3 mice; STRV, N = 2 mice. Source data are provided as a Source Data file.

Fig. S6

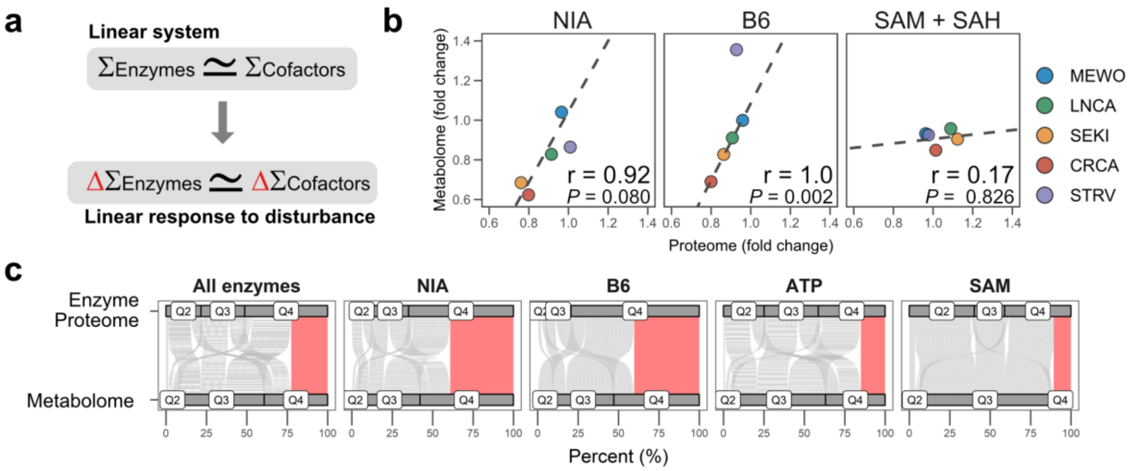

Fig. S6: A linear decrease of metabolites and enzymes related to NIA or B6 occurs in livers of mice with cancer cachexia. a, Schematic of linear response hypothesis deduced from the one-to-one relationship between cofactors and enzymes. Σ, data aggregation; ≃, approximately equal; red Δ, small change. **b,** Scatterplots of changes in NIA or B6-related metabolome and enzyme proteome. Dashed lines are produced by linear regression without the STRV point. r, Pearson`s correlation coefficient; *P, P* value (Pearson's correlation). **c,** Alluvial plot showing connections between the liver metabolome and enzyme proteome. Q, simple quarter, not quartile. Red streams indicate mapping between highly abundant enzymes (Q4) and metabolites (Q4). Streams of Q1s are not shown due to the small numbers. "All enzymes" signifies all analyzable enzymes; NIA, niacin; B6, B6 vitamin; PA, pantothenic acid. In many cases, the mapping from Q1 to Q1 failed. In B2 vitamin, the number of quantified substrates is too small for mapping. All enzymes, total streams (N = 2679 streams), Q4-Q4 streams (N = 603 streams); NIA, total streams (N = 727 streams), Q4-Q4 streams (N = 285 streams); B6, total streams (N = 174 streams), Q4-Q4 streams (N = 70 streams); ATP, total streams (N = 1323 streams), Q4-Q4 streams (N = 199 streams); SAM, total streams (N = 346 streams), Q4-Q4 streams (N = 37 streams). Source data are provided as a Source Data file.

Fig. S7

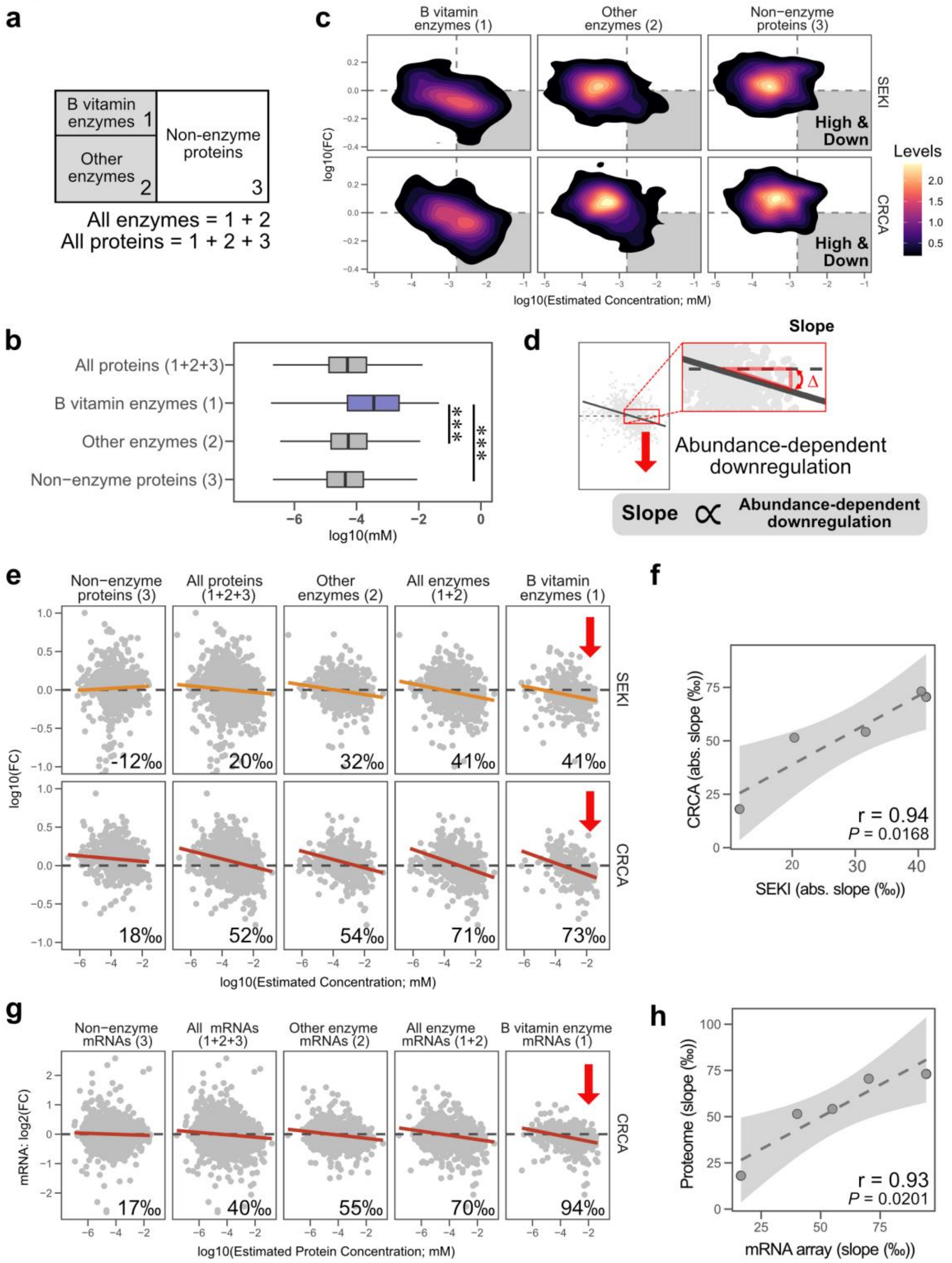

Fig. S7: Abundance-dependent hepatic proteomic changes in cancer cachexia. a, Schematic of three mutually exclusive proteome subsets based on UniProt annotations (Table S5). **b,** Box plots of protein concentrations estimated from MaxLFQ values of mouse liver by LC-MS^2. All proteins (1+2+3), 8645 proteins; B vitamin enzymes (1), 615 proteins; other enzymes (2), 2225 proteins; non-enzyme proteins (3), 5805 proteins.

Non-adjusted Welch's 2-sided *t*-test of log transformed data; B vitamin enzymes (1) vs other enzymes (2), $P = 2.2 \times 10^{-16}$; B vitamin enzymes (1) vs non-enzyme proteins (3), $P = 2.2 \times 10^{-16}$. ***$P < 0.001$. Boxplot style, the middle line (median), the upper hinge (Q3, the third quantile), the lower hinge (Q1, the first quantile), the upper whisker (1.5 × (Q3 – Q1)), the lower whisker (1.5 × (Q3 – Q1)). **c,** Density plots of SEKI and CRCA hepatic proteome datasets. Facet subtitles by Fig. S7a. High & Down, protein concentration above the mean of combined SEKI and CRCA hepatic proteome datasets and with a fold change less than 1. SEKI, N = 3 mice; CRCA, N = 3 mice. Protein concentrations were estimated from MaxLFQ values of mouse liver by LC-MS^2. Note that only proteins with an FC greater than 0.1 and less than 10 were analyzed and presented. Plots are limited to a range from -5 to -1 on the X-axis and -1 to 1 on the Y-axis. **d,** Schematic diagram showing the relationship between declining slope (Δ) and abundance-dependent downregulation. **e,** Scatterplots of SEKI and CRCA hepatic proteome datasets. SEKI, N = 3 mice; CRVA, N = 3 mice. FC, fold change. Colored, solid lines represent linear regression lines. Protein concentrations were estimated from the MaxLFQ value of mouse liver by LC-MS^2. Note that only proteins with an FC greater than 0.01 and less than 100 were analyzed and presented. Facet subtitles by Fig. S7a. Facet number, depression slope (permille); red arrows, massive decline of highly abundant B vitamin-related enzymes. Elevation slope is negative. Note that only proteins with an FC greater than 0.1 and less than 10 were presented. **f,** Scatterplot of absolute depression slopes presented in Fig. S7e. The dashed line was produced by linear regression. The gray zone indicates the 95% confidence interval around the dashed line. Abs., absolute; r, Pearson`s correlation coefficient; *P, P* value (Pearson's correlation). Pearson's correlation coefficient was used because slope values were distributed over a narrow range. **g,** Scatterplots of changes (log 2) in the CRCA liver transcriptome with published mouse protein abundance data on the X axis. CRCA, N = 4 mice; C57BL/6N, N =4 mice. FC, fold change. Red solid lines represent linear regression lines. Facet subtitles by Fig. S7a. Protein concentrations were estimated from MaxLFQ values of mouse liver by LC-MS^2. Facet number, depression slope (permille); red arrow, massive decline of highly abundant B vitamin-related enzyme mRNAs. **h,** Scatterplot of depression slopes presented in Fig. S7e and g. The dashed line is produced by linear regression. The gray zone indicates 95% confidence interval around the dashed line. r, Pearson`s correlation coefficient; *P, P* value (Pearson's correlation). Pearson's correlation coefficient was used because the slope values were distributed over a narrow range. Source data are provided as a Source Data file.

Fig. S8

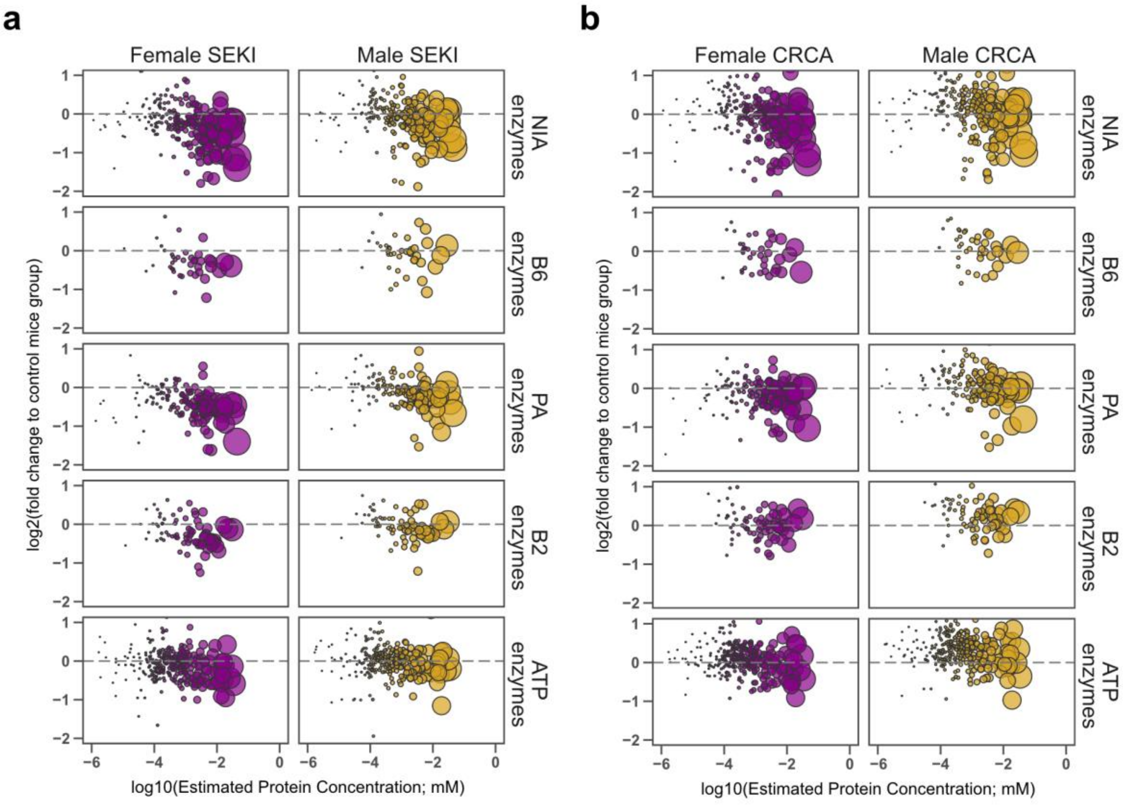

Fig. S8: No substantial sex-related differences in the declining trend of B-vitamin enzymes in SEKI and CRCA livers. a & b, Displays based on sex. Female SEKI, N = 3 mice; male SEKI, N = 3 mice; female CRCA, N = 3 mice; male CRCA, N = 3 mice. Bubble size represents protein concentration. MaxLFQ values were used to estimate protein concentrations[2]. Fold changes are from TMT quantitative proteomic analysis. NIA, niacin related enzymes; B6, vitamin B6 related enzymes; PA, pantothenic acid related enzymes; B2, vitamin B2 related enzymes; ATP, ATP related enzymes. The plots are limited to a range of -6 to 0 on the X-axis and -2 to 1 on the Y-axis. Source data are provided as a Source Data file.

Fig. S9

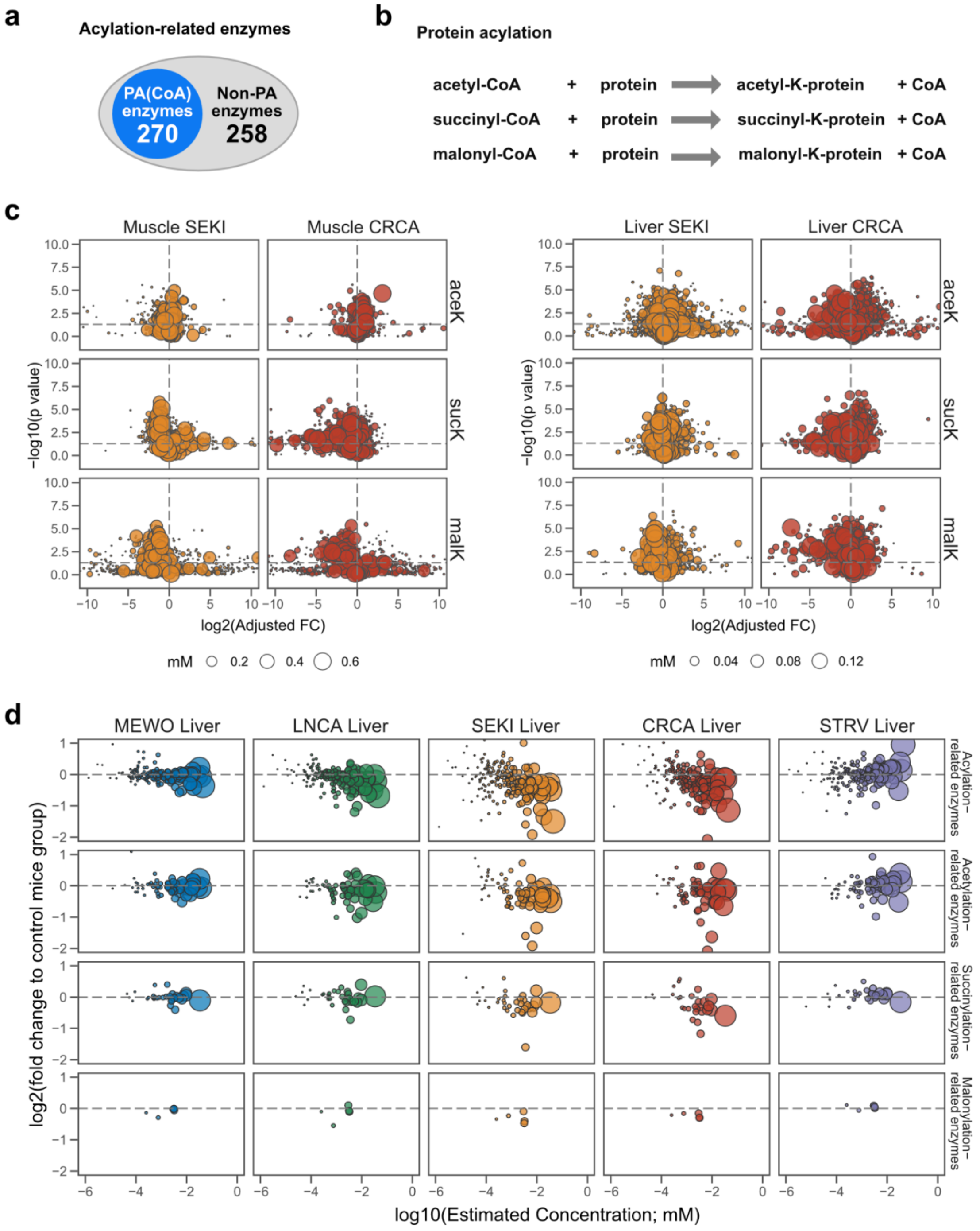

Fig. S9: Protein lysine malonylation is decreased in cachectic muscle. a, Venn diagram showing the relationship between acyl-related enzymes and PA (CoA)-related enzymes. PA, pantothenate (vitamin B5); CoA, coenzyme A. **b,** Schematic of protein acylation. K, lysine. **c,** Volcano plots of proteomic data for muscle and liver acetyl-K (aceK), succinyl-K (suck), and malonyl-K (malK). Bubble size, protein concentration estimated from published proteome data [2,3]. Adjusted FC, fold change adjusted by total

protein fold change. Horizontal dashed lines show where P = 0.05 (Welch's 2-sided *t*-test). Plots are limited to a range from -10 to 10 on the X-axis and -1 to 10 on the Y-axis. **d,** Keyword-based bubble plots of liver proteomic data. Bubble size represents protein concentration. MEWO, N = 3 mice; LNCA, N = 3 mice; SEKI, N = 3 mice; CRCA, N = 3 mice; STRV, N = 2 mice. MaxLFQ values were used to estimate protein concentrations[2]. Fold changes are from TMT quantitative proteomic analysis. Plots are limited to a range from -6 to 0 on the X-axis and -2 to 1 on the Y-axis. Source data are provided as a Source Data file.

Fig. S10

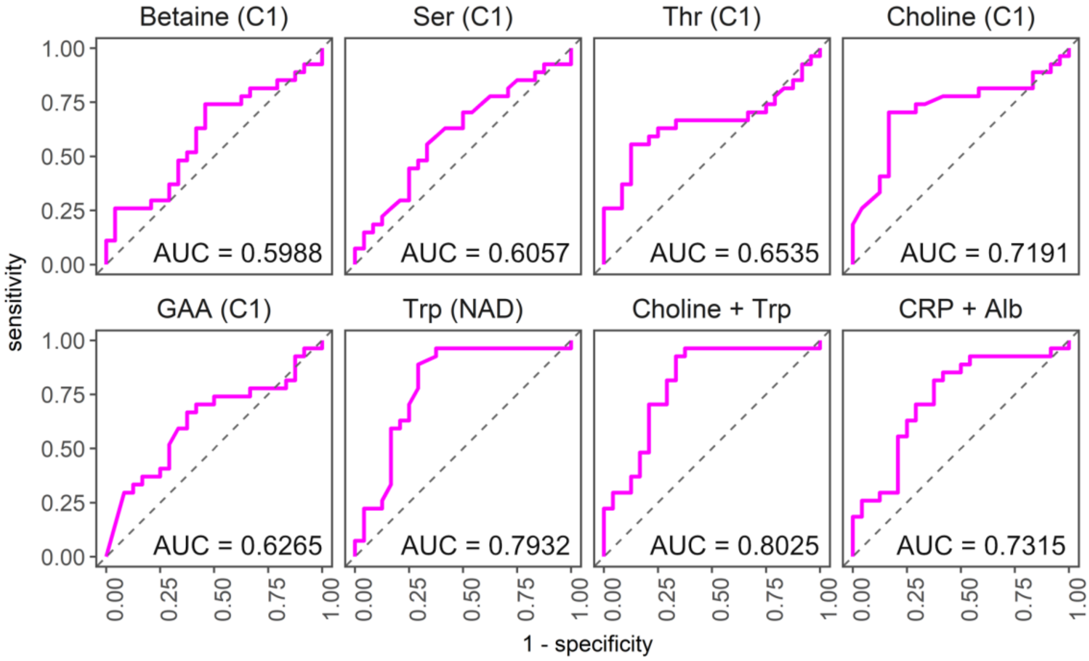

Fig. S10: Receive-operating characteristic (ROC) curves for discrimination of patients with or without cachexia. Gastric cancer patients were diagnosed for cachexia as previously reported (cachexia, N = 27 patients; non cachexia, N = 24 patients)[4]. AUC, area under curve; Ser, serine; Thr, threonine; GAA, guanidinoacetate; Trp, tryptophan; Choline + Trp, multivariate logistic modeling with choline and Trp fold-changes; CRP + Alb, multivariate logistic modeling with C-reactive protein (CRP) and albumin (Alb) concentrations. Source data are provided as a Source Data file.

Fig. S11

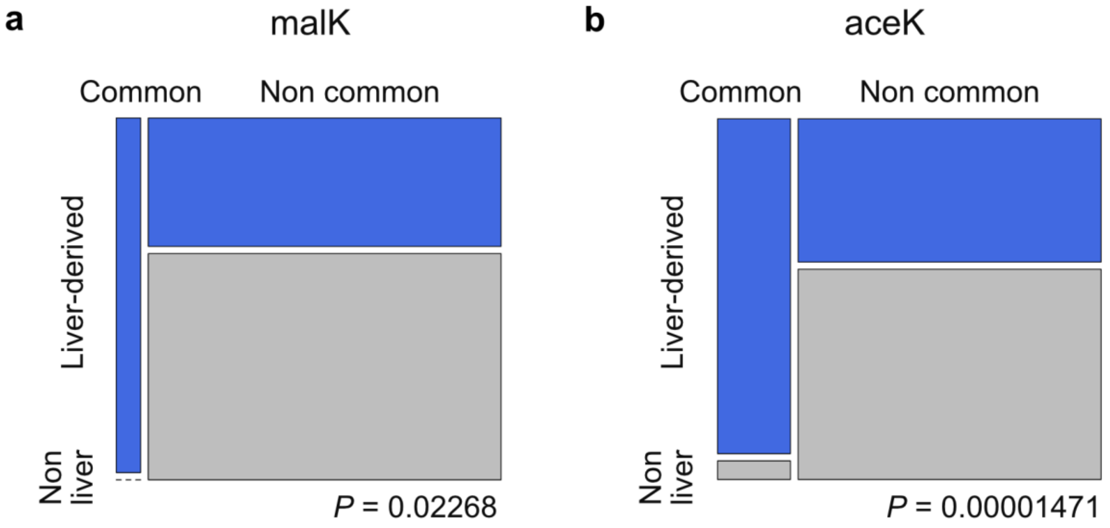

Fig. S11: Most malonylated and acetylated proteins commonly detected in mouse and human plasma are liver-derived. a & b, Mosaic plots of blood malonyalted (malK) and acetylated (aceK) proteins. Liver-derived, blood proteins derived from the liver (blue); Non liver, blood proteins not derived from the liver (grey); Common, commonly detected in mouse and human plasma; Uncommon, not commonly detected in mouse and human plasma. Two *P* values by Fisher's exact test. Source data are provided as a Source Data file.

Table S1: Mouse models

Abbreviation	Strain/Procedure	Background	Details
MEWO	Mewo xenograft mouse	BALB/c nu/nu	Subcutaneous inoculation of the human melanoma cell line Mewo (MEWO)
LNCA	*K-ras*$^{LSL\text{-}G12D/+}$/*p53*$^{lox/lox}$ mouse	C57BL/6[a]	Conditional lung cancer (LNCA) model using adenoviral delivery of Cre recombinase via the respiratory tract
SEKI	SEKI xenograft mouse	BALB/c nu/nu	Subcutaneous inoculation of the human melanoma cell line SEKI
CRCA	*cis-Apc*$^{+/\Delta716}$/*Smad4*$^{+/-}$ mouse	C57BL/6N	Spontaneous invasive colon cancer (CRCA) model
STRV	Mouse with 48-hour starvation	C57BL/6N	Fasted with food withdrawn and ad libitum water drinking for 48 hours

[a] The C57BL/6J lines were crossed several times with C57BL/6N.

Table S2: Welch's 2-sided *t*-test for Figs. 2a, 2b, 2e, 2f, S4a, and S4c

Figure	Group	Tissue	Metabolite	Non-adjusted *P* value	95% CI lower	95% CI upper
Figs. 2a & S4a	MEWO	Muscle	NAD	0.055283	-0.40916	0.005886
Figs. 2a & S4a	LNCA	Muscle	NAD	4.94E-05	-0.24974	-0.10387
Figs. 2a & S4a	SEKI	Muscle	NAD	0.001464	-0.59767	-0.17337
Figs. 2a & S4a	CRCA	Muscle	NAD	5.91E-07	-0.42968	-0.2269
Fig. 2a	STRV	Muscle	NAD	0.9938	-0.20457	0.206076
Fig. S4a	FK866	Muscle	NAD	0.037017	-0.5186	-0.01864
Fig. S4a	5FU	Muscle	NAD	0.009823	-0.4269438	-0.09524
Figs. 2a & S4a	MEWO	Liver	NAD	0.019547	-0.66511	-0.07311
Figs. 2a & S4a	LNCA	Liver	NAD	0.067993	-0.39995	0.015469
Figs. 2a & S4a	SEKI	Liver	NAD	0.003855	-0.98065	-0.23587
Figs. 2a & S4a	CRCA	Liver	NAD	1.72E-12	-0.63312	-0.4522
Fig. 2a	STRV	Liver	NAD	0.054521	-0.55985	0.006346
Fig. S4a	FK866	Liver	NAD	9.27E-07	-0.84749	-0.46233
Fig. S4a	5FU	Liver	NAD	0.275103	-0.22312	0.073484
Fig. 2a	MEWO	Liver	NADP	0.161568	-0.48265	0.094611
Fig. 2a	LNCA	Liver	NADP	0.006616	-0.31638	-0.05886
Fig. 2a	SEKI	Liver	NADP	3.25E-05	-0.70907	-0.32793
Fig. 2a	CRCA	Liver	NADP	2.03E-14	-0.58339	-0.42862
Fig. 2a	STRV	Liver	NADP	0.937467	-0.29369	0.272823
Fig. 2a	MEWO	Liver	NAM	0.014421	0.123609	0.736102
Fig. 2a	LNCA	Liver	NAM	0.850257	-0.31635	0.262893
Fig. 2a	SEKI	Liver	NAM	0.008265	-0.35644	-0.06188
Fig. 2a	CRCA	Liver	NAM	0.02969	-0.34019	-0.01885
Fig. 2a	STRV	Liver	NAM	0.282896	-0.34519	0.1093
Figs. 2b & S4a	MEWO	Muscle	NIA	0.096352	-0.23604	0.022833
Figs. 2b & S4a	LNCA	Muscle	NIA	0.000375	-0.20606	-0.06994
Figs. 2b & S4a	SEKI	Muscle	NIA	0.132845	-0.15259	0.023928
Figs. 2b & S4a	CRCA	Muscle	NIA	4.06E-07	-0.3932	-0.21095
Fig. 2b	STRV	Muscle	NIA	0.735681	-0.05651	0.077575
Fig. S4a	FK866	Muscle	NIA	0.008967	-0.43063	-0.0759

Fig. S4a	5FU	Muscle	NIA	0.000553	-0.28828	-0.1488
Figs. 2b & S4a	MEWO	Liver	NIA	0.560215	-0.15405	0.261917
Figs. 2b & S4a	LNCA	Liver	NIA	0.001876	-0.26251	-0.06807
Figs. 2b & S4a	SEKI	Liver	NIA	6.80E-08	-0.38814	-0.2315
Figs. 2b & S4a	CRCA	Liver	NIA	3.52E-17	-0.42061	-0.32799
Fig. 2b	STRV	Liver	NIA	0.001632	-0.20364	-0.06173
Fig. S4a	FK866	Liver	NIA	8.63E-07	-0.59206	-0.36279
Fig. S4a	5FU	Liver	NIA	0.026729	0.015594	0.189821
Figs. 2b & S4a	MEWO	Muscle	B6	0.308971	-0.82103	0.306298
Figs. 2b & S4a	LNCA	Muscle	B6	0.966671	-0.23414	0.243889
Figs. 2b & S4a	SEKI	Muscle	B6	0.563534	-0.16754	0.289961
Figs. 2b & S4a	CRCA	Muscle	B6	0.001335	-0.41337	-0.10936
Fig. 2b	STRV	Muscle	B6	0.671868	-0.24628	0.164757
Fig. S4a	FK866	Muscle	B6	0.575929	-0.14278	0.249106
Fig. S4a	5FU	Muscle	B6	0.693596	-0.3214	0.227242
Figs. 2b & S4a	MEWO	Liver	B6	0.886259	-0.2232	0.254624
Figs. 2b & S4a	LNCA	Liver	B6	0.099009	-0.17524	0.016253
Figs. 2b & S4a	SEKI	Liver	B6	0.002373	-0.26986	-0.06955
Figs. 2b & S4a	CRCA	Liver	B6	3.10E-09	-0.3854	-0.22643
Fig. 2b	STRV	Liver	B6	9.86E-05	0.217415	0.506124
Fig. S4a	FK866	Liver	B6	9.75E-05	-0.42822	-0.18106
Fig. S4a	5FU	Liver	B6	0.269639	-0.28377	0.091047
Fig. 2e	MEWO	Muscle	Betaine	0.018008	0.055277	0.452059
Fig. 2e	LNCA	Muscle	Betaine	0.092234	-0.02272	0.276969
Fig. 2e	SEKI	Muscle	Betaine	0.673856	-0.26863	0.178398
Fig. 2e	CRCA	Muscle	Betaine	0.009959	-0.28298	-0.04191
Fig. 2e	STRV	Muscle	Betaine	3.63E-09	-0.54932	-0.39288
Fig. 2e	MEWO	Liver	Betaine	0.051658	-0.00819	1.674247
Fig. 2e	LNCA	Liver	Betaine	0.083533	-0.05193	0.774356
Fig. 2e	SEKI	Liver	Betaine	0.194794	-0.3879	0.084111
Fig. 2e	CRCA	Liver	Betaine	1.91E-07	-0.6889	-0.36092
Fig. 2e	STRV	Liver	Betaine	0.029677	-0.75754	-0.04818

Figs. 2e & S4a	MEWO	Muscle	Gly	0.012749	-0.41708	-0.06775
Figs. 2e & S4a	LNCA	Muscle	Gly	0.002443	-0.49576	-0.12311
Figs. 2e & S4a	SEKI	Muscle	Gly	0.013606	-0.43365	-0.06027
Figs. 2e & S4a	CRCA	Muscle	Gly	8.96E-11	-0.65434	-0.41933
Fig. 2e	STRV	Muscle	Gly	4.97E-11	-0.54538	-0.43003
Fig. S4a	FK866	Muscle	Gly	0.064598	-0.45484	0.014609
Fig. S4a	5FU	Muscle	Gly	0.000146	-0.66305	-0.37763
Figs. 2e & S4a	MEWO	Liver	Gly	0.674108	-0.12008	0.177728
Figs. 2e & S4a	LNCA	Liver	Gly	0.474992	-0.21664	0.105766
Figs. 2e & S4a	SEKI	Liver	Gly	0.001045	-0.41607	-0.12227
Figs. 2e & S4a	CRCA	Liver	Gly	1.18E-13	-0.47433	-0.34207
Fig. 2e	STRV	Liver	Gly	0.005222	-0.38699	-0.09181
Fig. S4a	FK866	Liver	Gly	0.006289	-0.5928	-0.11476
Fig. S4a	5FU	Liver	Gly	0.009656	-0.48391	-0.10832
Fig. 2f	MEWO	Muscle	SAM	0.034877	-0.24183	-0.011
Fig. 2f	LNCA	Muscle	SAM	0.188913	-0.29596	0.062181
Fig. 2f	SEKI	Muscle	SAM	0.000661	-0.42049	-0.16116
Fig. 2f	CRCA	Muscle	SAM	3.69E-09	-0.44831	-0.29463
Fig. 2f	STRV	Muscle	SAM	1.01E-09	-0.43088	-0.3196
Fig. 2f	MEWO	Liver	SAM	0.017435	-0.76783	-0.09326
Fig. 2f	LNCA	Liver	SAM	2.06E-07	-0.72864	-0.40694
Fig. 2f	SEKI	Liver	SAM	0.758989	-0.29317	0.217004
Fig. 2f	CRCA	Liver	SAM	0.277356	-0.08474	0.283894
Fig. 2f	STRV	Liver	SAM	0.00916	-0.66729	-0.11542
Fig. 2f	MEWO	Muscle	SAH	0.228465	-0.62487	2.225532
Fig. 2f	LNCA	Muscle	SAH	0.998563	-1.00486	1.006622
Fig. 2f	SEKI	Muscle	SAH	0.018252	-0.48156	-0.05159
Fig. 2f	CRCA	Muscle	SAH	0.000205	-0.40873	-0.14687
Fig. 2f	STRV	Muscle	SAH	0.000226	-0.53838	-0.21181
Fig. 2f	MEWO	Liver	SAH	0.323633	-0.18584	0.506199
Fig. 2f	LNCA	Liver	SAH	0.000191	0.260622	0.676046
Fig. 2f	SEKI	Liver	SAH	0.327713	-0.35541	0.124284

Fig. 2f	CRCA	Liver	SAH	9.55E-08	-0.4264	-0.22846
Fig. 2f	STRV	Liver	SAH	0.164255	-0.1131	0.603518
Fig. S4a	MEWO	Muscle	SAM/SAH	0.143195	-0.84767	0.143601
Fig. S4a	LNCA	Muscle	SAM/SAH	0.755627	-0.7132	0.524765
Fig. S4a	SEKI	Muscle	SAM/SAH	0.967595	-0.3762	0.390577
Fig. S4a	CRCA	Muscle	SAM/SAH	0.252968	-0.27956	0.078439
Fig. S4a	STRV	Muscle	SAM/SAH	0.718557	-0.20519	0.287924
Fig. S4a	FK866	Muscle	SAM/SAH	0.251029	-0.11507	0.413245
Fig. S4a	5FU	Muscle	SAM/SAH	0.026109	-0.38664	-0.0321
Fig. S4a	MEWO	Liver	SAM/SAH	0.082858	-1.06347	0.080239
Fig. S4a	LNCA	Liver	SAM/SAH	3.00E-06	-0.93863	-0.5156
Fig. S4a	SEKI	Liver	SAM/SAH	0.586344	-0.33837	0.580857
Fig. S4a	CRCA	Liver	SAM/SAH	0.004892	0.187033	0.916248
Fig. S4a	STRV	Liver	SAM/SAH	0.023486	-0.93162	-0.08065
Fig. S4a	FK866	Liver	SAM/SAH	0.001716	0.652861	2.22218
Fig. S4a	5FU	Liver	SAM/SAH	0.010652	0.308014	1.694302
Fig. S4c	MEWO	Liver	MNA	0.462381614	-0.192995057	0.392186527
Fig. S4c	LNCA	Liver	MNA	0.892235409	-0.284232577	0.324426363
Fig. S4c	SEKI	Liver	MNA	6.97E-06	1.025383686	2.001392498
Fig. S4c	CRCA	Liver	MNA	1.52E-07	0.734761789	1.2558784
Fig. S4c	STRV	Liver	MNA	0.007231216	-0.324158952	-0.061100126
Fig. S4c	MEWO	Liver	PTRS	0.135238199	-0.109153411	0.652597127
Fig. S4c	LNCA	Liver	PTRS	0.004450735	0.189604272	0.845973428
Fig. S4c	SEKI	Liver	PTRS	2.52E-07	1.801054871	2.810268383
Fig. S4c	CRCA	Liver	PTRS	6.92E-05	7.956684412	17.49726688
Fig. S4c	STRV	Liver	PTRS	0.915844559	-1.504760352	1.663588998

NAM, nicotinamide; MNA, 1-methyl nicotinamide; PTRS, putrescine.
Source data are provided as a Source Data file.

Table S3: Steel's 1-sided test for LNCA*, SEKI and CRCA groups

Figure	Metabolite	Tissue	Statistic	Non-adjusted *P* value	Alternative
Fig. 2a	NAD	muscle	-3.099605208	0.002090169	less
Fig. 2a	NAD	liver	-3.513078076	0.000492868	less
Fig. 2a	NADP	liver	-3.50516103	0.000500332	less
Fig. 2a	NAM	liver	-1.608792764	0.100239799	less
Fig. 2b	NIA	muscle	-3.2154783	0.001413698	less
Fig. 2b	B6	muscle	-2.520239749	0.012155744	less
Fig. 2b	NIA	liver	-3.910716852	0.000102766	less
Fig. 2b	B6	liver	-4.084526489	4.97E-05	less
Fig. 2e	Betaine	muscle	-3.273414846	0.001156993	less
Fig. 2e	Gly	muscle	-2.578176295	0.010343238	less
Fig. 2e	Betaine	liver	-4.084526489	4.97E-05	less
Fig. 2e	Gly	liver	-4.142463035	3.87E-05	less
Fig. 2f	SAM	muscle	-3.331351392	0.000943834	less
Fig. 2f	SAH	muscle	0.997042647	0.946229357	less
Fig. 2f	SAM / SAH	muscle	-0.492460641	0.476449247	less
Fig. 2f	SAM	liver	4.084526489	4.97E-05	greater
Fig. 2f	SAH	liver	-4.142463035	3.87E-05	less
Fig. 2f	SAM / SAH	liver	4.142463035	3.87E-05	greater
Fig. S4c	MNA	liver	3.968653398	8.09E-05	greater
Fig. S4c	PTRS	liver	4.142463035	3.87E-05	greater

* Control group; MNA, 1-methyl nicotinamide; PTRS, putrescine

Table S4: Dunnett's 2-sided test for LNCA*, SEKI and CRCA groups for Fig. 2b

term	contrast	Adjusted *P* value	95% CI Lower Bound	95% CI Upper Bound
Liver NIA	SEKI - LNCA	0.001152451	-0.232199292	-0.056854617
Liver NIA	CRCA - LNCA	4.63E-06	-0.293244196	-0.124778482
Liver B6	SEKI - LNCA	0.019784361	-0.167012409	-0.01340688
Liver B6	CRCA - LNCA	9.55E-08	-0.300209246	-0.152629828

* Control group; Source data are provided as a Source Data file.

Table S5: List of Keywords and Search Formula for Data Aggregation

Object	Metabolite (CE-MS; KEGG Compound ID)	Metabolite (Food Composition*)	Protein (UniProtKB; UniProt ID, EC number, Catalytic activity, Cofactor, Keyword ID, Nucleotide binding, ChEBI)
**Acetylation-related enzymes**	NA	NA	("Acetyl" OR "acetyl" OR "acetate" OR "acetic acid") AND (NOT ("N-acetyl") AND (NOT ("acetate" OR "acetic acid")))
**Acyl**	NA	NA	“CoA” OR “KW-0173” OR “acyl”
**All enzymes**	NA	NA	(NOT (Catalytic activity = “NA”)) OR (NOT (EC number = “NA”))
**Apolipoproteins**	NA	NA	"KW-0427” OR “KW-0345” OR “KW-0162"
**ATP enzymes**	NA	NA	“ATP” OR “KW-0067”
**B vitamins**	NA	NA	NIA OR B6 OR B2 OR PA OR B1 OR B12 OR Folate
**B1**	“C00378” OR “C01081”	Thiamin (Fluorescence detection)	“thiamine” OR (“KW-0786”)
**B12**	Mutti E. et al., 2013: mouse hepatic cobalamins (Cbls), 443.09 pmol/g	Vitamin B_{12} (Microbiological assay)	“methylcob” OR “KW-0170” OR “KW-0846”
**B2**	“C00016”	Riboflavin (Fluorescence detection)	“FAD” OR “FMN” OR “KW-0274” OR “KW-0288” OR “KW-0285” OR “KW-0013”
**B6**	“C00647” OR “C00250”	Vitamin B_6 (Microbiological assay)	“pyridoxal” OR “pyridoxal” OR “KW-0663” OR “KW-0032” OR “KW-0664”
**Biotin**	Yuasa M. et al., 2016: Table 1; rat hepatic biotin, 7.4 nmol/g protein.	Biotin (Microbiological assay)	“biotin” OR “KW-0092”
**C1**	NA	NA	“glycine” OR “serine” OR “S-adenosyl-L-methionine” OR “folate” OR “KW-0489” OR “KW-0554” OR “KW-0486” OR “KW-0658” OR “KW-0660” OR “KW-0545” OR “KW-0198” OR “KW-0290”
**Endoplasmic reticulum**	NA	NA	“KW-0256”
**Folate**	Bills, et al., 1992: Table 2; mouse hepatic folate, 9.263 µg/g	Folate (Microbiological assay)	“folate” OR “KW-0290”
**Gly enzymes**	NA	NA	“CHEBI:57305;”

**Golgi**	NA	NA	“KW-0333”
**Immunity including APPs**	NA	NA	“KW-0395” R “KW-0391” OR “KW-0399” OR “KW-1064” OR “KW-1280” OR “KW-0011” OR “KW-0180” OR “KW-0179” OR KW-1018" OR "CO3_” OR “CO4A_” OR “CO4B_” OR “AACT_” OR “FIBB_”, OR “FIBA_” OR “CERU_"
**Liver-derived proteins**	NA	NA	Secretory proteins presumed to be of liver origin (Franko et al., 2019)
**Malonylation-related enzymes**	NA	NA	("Malonyl" OR "malonyl" OR "malonate" OR "malonic acid") AND (NOT ("methylmalonyl" OR "Methylmalonyl"))
**Mitochondria**	NA	NA	“KW-0496”
**NIA**	“C00003” OR “C00004” OR “C00005” OR “C00006” OR “C00153”	Niacin (Microbiological assay)	“NAD” OR “NADP” OR “nicoti” OR “KW-0520” OR “KW-0521” OR “KW-0662” OR “KW-0013”
**Non-enzyme proteins**	NA	NA	NOT (All enzymes)
**PA**	“C00864” OR “C00010” OR “C00024” OR “C00630” OR “C00099”	Pantothenic acid (Microbiological assay)	“CoA” OR “KW-0173”
**Polyamines**	“C00134” OR “C00750” OR “C00315” OR “C00612” OR “C00170”	NA	NA
**Positive APPs**	NA	NA	“KW-0011” OR "CO3_” OR “CO4A_” OR “CO4B_" OR "AACT_” OR “FIBB_” OR “FIBA_” OR “CERU_"
**Ribosome**	NA	NA	“KW-0687”
**SAM enzymes**	NA	NA	“S-adenosyl-L-methionine” OR "spermidine" OR "spermine", OR "putrescine" OR "polyamine" OR "L-methionine" OR "L-homocysteine"
**Succinylation-related enzymes**	NA	NA	"Succinyl" OR "succinyl" OR "succinate" OR "succinic acid"

* Standard Tables of Food Composition in Japan -2015- (Seventh Revised Edition)

Table S6: Baseline Characteristics of Patients

**Characteristic**	**Early gastric cancer (n = 16)**	**Advanced gastric cancer (n = 41)**	**Non-adjusted *P* value**
Median age (range)	62 (51-73)	67 (40-80)	Welch 0.0258
Sex			Fisher 0.753
Female	4	13	
Male	12	28	
Stage			
Stage I and II cancer	16 (100%)	0 (0%)	
Stage IV cancer	0 (0%)	41(100%)	
Gastrointestinal obstruction	0/16 (0%)	9/32 (28%)	Fisher 0.04953
Cancer Cachexia*	2/16 (13%)	25/30 (83%)	Fisher 1.61×10^{-4}
Mean BMI (range)	22.9 (18.0-29.1)	19.2 (13.4-25.4)	Welch 8.56×10^{-5}
Mean serum albumin (g/L)	43 (37-48)	28 (18-40)	Welch 2.2×10^{-16}
Mean serum CRP (mg/L)	0.8 (0.1-6.6)	34.8 (0.6-202.5)	Welch 3.85×10^{-5}
Glasgow Prognostic Score**			
GPS = 0	16 (100%)	4 (10%)	
GPS = 1	0 (0%)	10 (24%)	
GPS = 2	0 (0%)	27 (66%)	

BMI, body mass index; CRP, C-reactive protein; Welch, Welch's 2-sided *t*-test; Fisher, Fisher's exact test.
*Based on the diagnostic criteria for cancer cachexia by Fearon et al[4]. Only patients whose weight change could be tracked in the medical records of Aichi Cancer Center.
**Based on Forrest et al[5].

Supplementary References

1. Geiger, T. *et al.* Initial quantitative proteomic map of 28 mouse tissues using the SILAC mouse. *Mol. Cell. Proteomics* **12**, 1709–1722 (2013).
2. Azimifar, S. B., Nagaraj, N., Cox, J. & Mann, M. Cell-type-resolved quantitative proteomics of murine liver. *Cell Metab.* **20**, 1076–1087 (2014).
3. Wang, M., Herrmann, C. J., Simonovic, M., Szklarczyk, D. & von Mering, C. Version 4.0 of PaxDb: Protein abundance data, integrated across model organisms, tissues, and cell-lines. *Proteomics* **15**, 3163–3168 (2015).
4. Fearon, K. *et al.* Definition and classification of cancer cachexia: an international consensus. *Lancet Oncol.* **12**, 489–495 (2011).
5. Forrest, L. M., McMillan, D. C., McArdle, C. S., Angerson, W. J. & Dunlop, D. J. Evaluation of cumulative prognostic scores based on the systemic inflammatory response in patients with inoperable non-small-cell lung cancer. *Br. J. Cancer* **89**, 1028–1030 (2003).
