# Supplementary material for: Decreased liver B vitamin-related enzymes as a metabolic hallmark of cancer cachexia
Source: Nat Commun. 2023 Oct 6;14:6246. doi: 10.1038/s41467-023-41952-w (PMC10558488; doi:10.1038/s41467-023-41952-w)
Supplement: Supplementary file 3 — Reporting Summary [file 41467_2023_41952_MOESM3_ESM.pdf]

nature portfolio

Corresponding author(s): Masahiro Aoki and Yasushi Kojima

Last updated by author(s): Aug 22, 2023

Reporting Summary

Nature Portfolio wishes to improve the reproducibility of the work that we publish. This form provides structure for consistency and transparency in reporting. For further information on Nature Portfolio policies, see our Editorial Policies and the Editorial Policy Checklist.

Statistics

For all statistical analyses, confirm that the following items are present in the figure legend, table legend, main text, or Methods section.

n/a	Confirmed	
☐	☒	The exact sample size (*n*) for each experimental group/condition, given as a discrete number and unit of measurement
☐	☒	A statement on whether measurements were taken from distinct samples or whether the same sample was measured repeatedly
☐	☒	The statistical test(s) used AND whether they are one- or two-sided<br>*Only common tests should be described solely by name; describe more complex techniques in the Methods section.*
☒	☐	A description of all covariates tested
☒	☐	A description of any assumptions or corrections, such as tests of normality and adjustment for multiple comparisons
☐	☒	A full description of the statistical parameters including central tendency (e.g. means) or other basic estimates (e.g. regression coefficient) AND variation (e.g. standard deviation) or associated estimates of uncertainty (e.g. confidence intervals)
☐	☒	For null hypothesis testing, the test statistic (e.g. *F*, *t*, *r*) with confidence intervals, effect sizes, degrees of freedom and *P* value noted<br>*Give P values as exact values whenever suitable.*
☒	☐	For Bayesian analysis, information on the choice of priors and Markov chain Monte Carlo settings
☒	☐	For hierarchical and complex designs, identification of the appropriate level for tests and full reporting of outcomes
☐	☒	Estimates of effect sizes (e.g. Cohen's *d*, Pearson's *r*), indicating how they were calculated

Our web collection on statistics for biologists contains articles on many of the points above.

Software and code

Policy information about availability of computer code

Data collection	No new algorithms were developed for this manuscript.<br><br>MasterHands, version, 2.17.0.10, PMID: 20300169, https://pubmed.ncbi.nlm.nih.gov/20300169/<br>Mascot version 2.4, MATRIX SCIENCE, http://www.matrixscience.com/<br>Skyline version 20.1, MacCoss Lab, Biological Mass Spectrometry, University of Washington Genome Sciences Department, https://skyline.ms/project/home/software/Skyline/begin.view

Data analysis

No new algorithms were developed for this manuscript.
All the functions from R packages used in this study are stated in the Method section.

R version 4.1.3, R Foundation for Statistical Computing, https://www.r-project.org/
RStudio IDE, version 2022.07.01+554, Posit, PBC, https://posit.co/download/rstudio-desktop/
R package; limma, version 3.50.3; ggplot2, version 3.4.2; tidyverse, version 2.0.0; ggthemes, version, 4.2.4; viridis, version, 0.6.2; patchwork, version, 1.1.2; survminer, version, 0.4.9; PMCMRplus, version 1.9.7; multcomp, version 1.4-23; e1071, version 1.7.13; randomForest, version 4.7-1.1; ggcorrplot, version 0.1.4; car, version 3.1.2; survival, version, 3.2-13; clinfun, version, 1.1.1; pROC version, 1.18.2

PyMOL version 2.5.2, Schrodinger, https://pymol.org/2/
Affnity Photo, version, 1.10.6.1665, https://affinity.serif.com/en-gb/photo/
ImageJ version 1.54d, https://imagej.nih.gov/ij/

a GO tool, https://agotool.org/

For manuscripts utilizing custom algorithms or software that are central to the research but not yet described in published literature, software must be made available to editors and reviewers. We strongly encourage code deposition in a community repository (e.g. GitHub). See the Nature Portfolio guidelines for submitting code & software for further information.

Data

Policy information about availability of data

All manuscripts must include a data availability statement. This statement should provide the following information, where applicable:

- Accession codes, unique identifiers, or web links for publicly available datasets
- A description of any restrictions on data availability
- For clinical datasets or third party data, please ensure that the statement adheres to our policy

The processed mouse and human metabolome data analyzed in this study are available as a Source Data: metabolome_cems.xlsx. The mouse and human proteome data generated in this study have been deposited in jPOST repository database (project ID, JPST001807, accession ID, PXD035832 [https://repository.jpostdb.org/entry/JPST001807]; project ID, JPST002183, accession ID, PXD042807 [https://repository.jpostdb.org/entry/JPST002183]). The mouse cachectic liver DNA microarray data used in this study have been deposited in the ArrayExpress database under accession code E-MTAB-11771 [https://www.ebi.ac.uk/biostudies/arrayexpress/studies/E-MTAB-11771].

Human Protein Atlas (liver expressed genes, N =981) reference dataset [https://www.proteinatlas.org/humanproteome/tissue/liver] downloaded in 2022 and used in this study is provided as a Source Data file (file name, reference_hpa.xlsx). The quantitative proteomic map of 28 mouse tissues using the SILAC mice re-analyzed in this study (Fig. S3d) is publicly available in PubMed Central under accession code PMC3675825 (file name, supp_M112.024919_mcp.M112.024919-2; GUID: BE9B5A17-41BF-442D-911B-DC3F6144F83B) [https://www.ncbi.nlm.nih.gov/pmc/articles/PMC3675825/]. The quantitative proteomics data of murine liver used in this study is publicly downloadable from the website of ScienceDirect through the PubMed under accession code PMID: 25470552 (table name, Table S1. Identified and Quantified Proteins in HCTs; file name, 1-s2.0 S1550413114004999-mmc2.xlsx) [https://www.sciencedirect.com/science/article/pii/S1550413114004999?via%3Dihub]. The Standards Tables of Food Composition in Japan 2015 (Seventh Revised Edition) Documentation and Table analyzed in this study (Fig. 4d, 4e, and 4f) are openly accessible on the website of the Ministry of Education, Culture, Sports, Science and Technology, Japan [https://www.mext.go.jp/en/policy/science_technology/policy/title01/detail01/1374030.htm]. The datasets of protein abundance used in this study are publicly available in the PAXdb (mouse liver, M.musculus – Liver (Integrated) [https://pax-db.org/dataset/10090/4150929682/]; S. cerevisiae, S.cerevisiae – Whole organism (Integrated) [https://pax-db.org/dataset/4932/1329501331/]; B. taurus, B.taurus – Whole organism (Integrated) [https://pax-db.org/dataset/9913/690473806/]; A. thaliana, A.thaliana – Whole organism (Integrated)[https://pax-db.org/dataset/3702/936750577/]; human plasma excluded, H.sapiens – Plasma (Integrated) [https://pax-db.org/dataset/9606/2315144209/]. The dataset of Rossmann-like domains (RLDs) re-analyzed in this study (Fig. 4g, 4h, and 4i) is publicly accessible in PubMed Central under accession PMC6957218 (S4 Table; GUID: 60A9D1A3-812B-4808-9F1B-834009B29B38) [https://www.ncbi.nlm.nih.gov/pmc/articles/PMC6957218/]. The datasets of crystal structure of human LDHA and NNMT presented in this study (Fig. 4g) are publicly available in the Protein Data Bank in Europe (PDBe) under the accession codes 5w8k [https://www.ebi.ac.uk/pdbe/entry/pdb/5w8k] and 2iip [https://www.ebi.ac.uk/pdbe/entry/pdb/2iip]. The dataset of secretory proteins presumed to be of liver origin used in this study (Fig. 8c and 8d) is publicly accessible in PubMed Central under accession PMC6723870 (file name, nutrients-11-01795-s001.zip; GUID, 5F20CF21-D0D2-4B1B-A930-DA335032CE21; table name, Table S2) [https://www.ncbi.nlm.nih.gov/pmc/articles/PMC6723870/]. Reference numbers of protein concentrations and tissue-specific gravity are freely available in the BioNumbers database under accession codes 113242 [https://bionumbers.hms.harvard.edu/bionumber.aspx?id=113242&ver=4&trm=113242&org=] and 115456 [https://bionumbers.hms.harvard.edu/bionumber.aspx?id=115456&ver=2&trm=115456&org=].

Source data are provided with this paper. The remaining data are available within the Article, Supplementary Information, or Source Data file.

Human research participants

Policy information about studies involving human research participants and Sex and Gender in Research.

Reporting on sex and gender

We only report results for sex; no information on gender has been collected. We analyzed 40 male and 17 female Japanese gastric cancer patients in this study. Gastric cancer is approximately twice as common in Japanese men as in Japanese women, which may explain the high number of male patients in our study. We did not recognize apparent sex differences in our data related to cancer cachexia. However, we can draw no definite conclusions regarding sex differences from this clinical data due to the small number of cases.

Population characteristics

The patient characteristics analyzed in this study are summarized in Table S6. We retrospectively analyzed frozen stocked human residual blood samples from Japanese patients with gastric cancer at Aichi Cancer Center Hospital (N = 57, strongly presumed to be of East Asian ethnicity). Diagnosis and clinical staging of gastric cancer was based on Japanese gastric cancer treatment guidelines 2014. We classified early gastric cancer and advanced cancer according to the clinical stage at the time

	of blood sampling (total n = 57; early gastric cancer, stage I and II, n = 16; advanced gastric cancer, stage III and IV, n = 41) and stratified patients into three groups using the classical Glasgow Prognostic Score (GPS = 0, n = 20; GPS = 1, n = 10; GPS = 2, n = 27)
Recruitment	We retrospectively analyzed residual cryopreserved blood samples collected from Japanese gastric cancer patients who gave written informed consent or were provided the opportunity to opt out at Aichi Cancer Center Central Hospital from January 2015 to December 2019. Diagnosis and clinical staging of gastric cancer were in accordance with the Japanese Guidelines for Gastric Cancer Practice 2014.
Ethics oversight	We obtained research ethics approvals from the ethics committee of Aichi Cancer Center Hospital (No. 2015-2-13; No. 2019-1-540).

Note that full information on the approval of the study protocol must also be provided in the manuscript.

Field-specific reporting

Please select the one below that is the best fit for your research. If you are not sure, read the appropriate sections before making your selection.

☒ Life sciences ☐ Behavioural & social sciences ☐ Ecological, evolutionary & environmental sciences

For a reference copy of the document with all sections, see nature.com/documents/nr-reporting-summary-flat.pdf

Life sciences study design

All studies must disclose on these points even when the disclosure is negative.

Sample size	No statistical method was used to predetermine sample size. We determined sample size empirically based on previous our studies (PMID: 19458066; PMID: 28847964; PMID: 36066360) and preliminary experimentation.
Data exclusions	No data exclusion criteria were set a priori. Before fixing the analysis data, we excluded some LC-MS-based proteomics data that we determined to be technical errors, including sample preparation failures (skeletal muscle and liver data from mouse fasting experiment, and human clinical blood data).
Replication	For mouse experiments, small pilot experiments were conducted before main experiments to optimize experimental conditions and evaluate general mouse conditions. The mouse phenotypes of the optimized pilot experiments were confirmed in the following main experiments.<br><br>Omics analyses in this study were performed with independent biological replicates. Regarding the biological replicates, their size was based on our previous studies19,82,83 and preliminary experimentation, and their details are described in the Methods section; Calculation of fold changes (FC) and sample numbers. The number of replicates is also stated in the figure legends. All the omics analyses with biological replicates and experimental controls produced acceptable and analyzable data in a single run. In addition, we validated each omics data against different mouse models or species data and literature-based knowledge. Therefore, we did not repeat omics experiments with some exceptions described below. We obtained additional SEKI and CRCA liver proteome data for sex-based analysis with new independent biological samples. The repeated results are roughly the same as the first time (Fig. 5a and Fig. S8). Regarding SEKI and CRCA metabolomics analyses, we performed small-scale experiments using stable metabolic isotopes; four times for the SEKI liver metabolome, twice for the SEKI muscle metabolome, four times for the CRCA liver metabolome, and four times for the CRCA muscle metabolome. The repeated omics runs yielded results broadly similar to the first run. This manuscript presents the first SEKI and CRCA metabolome data with the largest number of biological replicates.<br><br>The B-vitamin cocktail administration experiment using SEKI model was performed only once because the first experiment result was not significant (Fig. S3a; Cnt, N = 12 female mice; B-vitamin cocktail (Bvc), N = 12 female mice; Log rank test, P = 0.65). The glycine administration experiment was performed twice, and the data were combined. The first experiment result was significant (Cnt, N = 8 female mice; Gly, N = 8 female mice; Log rank test, P = 0.023), but not the second experiment result (Cnt, N = 16 female mice; Gly, N = 15 female mice; Log rank test, P = 0.29). Fig. S3b shows the combined results of the first and second experiments (Cnt, N = 24 female mice; Gly, N = 23 female mice; Log rank test, P = 0.021).
Randomization	Random allocation was performed in the mouse transplantation experiments, starvation experiments, and pharmacological experiments in which either glycine, B vitamin cocktail, 5FU, or FK866 was administrated. Where indicated in the data analysis, random sampling was performed using pseudo-random numbers.
Blinding	The metabolome and proteome measurements were performed in a blinded fashion. Blinding was not possible for the mouse experiments due to lack of human resources.

Reporting for specific materials, systems and methods

We require information from authors about some types of materials, experimental systems and methods used in many studies. Here, indicate whether each material, system or method listed is relevant to your study. If you are not sure if a list item applies to your research, read the appropriate section before selecting a response.

Materials & experimental systems

n/a	Involved in the study
☐	☒ Antibodies
☐	☒ Eukaryotic cell lines
☒	☐ Palaeontology and archaeology
☐	☒ Animals and other organisms
☒	☐ Clinical data
☒	☐ Dual use research of concern

Methods

n/a	Involved in the study
☒	☐ ChIP-seq
☒	☐ Flow cytometry
☒	☐ MRI-based neuroimaging

Antibodies

Antibodies used

Details of all the antibodies and antibody-based kits are stated in the Method section.

Antibodies for western blotting: Malonyl-Lysine [Mal-K] MultiMab Rabbit mAb mix (Cell Signaling Technology Cat#1492S; RRID: AB_2687627; lot number, 3; dilution, 1:1000), Anti-Malonyllysine Rabbit pAb (PTM BIO; Cat#PTM-901; lot number, #ZC0621105P0; dilution, 1:1000), Anti-Rabbit HRP-conjugated secondary antibody (Cell Signaling Technology Cat#7074; RRID: AB_2099233; lot number, not specified; dilution, 1:3000), Anti-GAPDH antibody conjugated with HRP (FUJIFILM Wako Cat#015-25473; RRID: AB_2665526; lot number, not specified; dilution, 1:10000).

Enzyme-linked immunosorbent assay (ELISA) kits; Mouse Serum Amyloid A Quantikine ELISA kit (R&D systems, Cat# MSAA00; lot number, not recorded; dilution, NA), Mouse Albumin ELISA kit (Abcam, Cat# ab207620; lot number, not recorded; dilution, NA), Mouse IL-6 ELISA kit (Merk, Cat#RAB0308: lot number, not recorded; dilution, NA), Mouse/Rat GDF-15 Quatikine ELISA kit (R&D systems, Cat#MGD150; lot number, not recorded; dilution, NA).

Antibody-based proteomics kits; PTMScan Acetyl-Lysine [Ac-K] Kit (Cat# 13416, Cell Signaling Technology; lot number, 6; dilution, NA), PTMScan Succinyl-Lysine [Succ-K] Kit (Cat# 13764, Cell Signaling Technology; lot number, 2; dilution, NA), PTMScan Malonyl-Lysine [Mal-K] Kit (Cat# 93872, Cell Signaling Technology; lot number, 1; dilution, NA).

Validation

All antibodies and antibody-based kits used in this study were commercially available and validated by manufacturers.

Antibodies for western blotting
1) Malonyl-Lysine [Mal-K] MultiMab Rabbit mAb mix, https://www.cellsignal.com/products/proteomic-analysis-products/malonyl-lysine-mal-k-multimab-rabbit-mab-mix/14942
2)Anti-Malonyllysine Rabbit pAb, https://ptmbio.com/products/anti-malonyllysine-rabbit-pab/PTM-901.htm
3) Anti-rabbit IgG, HRP-linked Antibody, https://www.cellsignal.com/products/secondary-antibodies/anti-rabbit-igg-hrp-linked-antibody/7074?site-search-type=Products&N=4294956287&Ntt=anti-rabbit+igg%2C+hrp-linked+antibody%2C&fromPage=plp&_requestid=430561
4) Anti GAPDH, Monoclonal Antibody, Peroxidase Conjugated (5A12), https://labchem-wako.fujifilm.com/asia/product/detail/W01W0101-2547.html

To further confirm the results of the PTM proteomics analyses (Fig. 7b), we planned western blotting analyses using malonyl-Lysine [Mal-K] MultiMab Rabbit mAb mix (Cell Signaling Technology Cat#1492S; RRID: AB_2687627; lot number, 3; dilution, 1:1000). As a pilot experiment, we initially analyzed SEKI and CRCA liver samples and observed downregulation of protein malonylation in the cachectic liver lysates (Source Data, Fig7d.xlsx, CST_validation_liver; BALB/c nu/nu, N = 4; SEKI, N = 4; C57BL/6N, N = 4; CRCA, N = 4). Next, we conducted another western blotting analysis using a different antibody, Anti-Malonyllysine Rabbit pAb (PTM BIO; Cat#PTM-901; lot number, #ZC0621105P0; dilution, 1:1000) and obtained roughly similar western blotting results, re-confirming downregulation of protein malonylation in the cachectic liver lysates (Source Data, Fig7d.xlsx, PTM_validation_liver; BALB/c nu/nu, N = 4; SEKI, N = 4; C57BL/6N, N = 4; CRCA, N = 4). Based on these validation experiments, we conducted the Fig. 7c's analyses (BALB/c nu/nu, N = 1; SEKI, N = 1; C57BL/6N, N = 1; CRCA, N = 1; C57BL/6N, N = 1; STRV, N = 1).

ELISA kits
1) Mouse Serum Amyloid A Quantikine ELISA kit, https://www.rndsystems.com/products/mouse-serum-amyloid-a-quantikine-elisa-kit_msaa00
2) Mouse Albumin ELISA kit, https://www.abcam.com/products/elisa/mouse-albumin-elisa-kit-ab207620.html
3) Mouse IL-6 ELISA kit, https://www.sigmaaldrich.com/JP/en/product/sigma/rab0308
4) Mouse/Rat GDF-15 Quatikine ELISA kit, https://www.rndsystems.com/products/mouse-rat-gdf-15-quantikine-elisa-kit_mgd150

Antibody-based proteomics kits (Cell Signaling Technology, https://www.cellsignal.com/)
1)PTMScan Acetyl-Lysine [Ac-K] Kit, https://www.cellsignal.com/products/proteomic-analysis-products/acetyl-lysine-motif-ac-k-kit/13416?site-search-type=Products&N=4294956287&Ntt=%2313416&fromPage=plp&_requestid=705263
2)PTMScan Succinyl-Lysine [Succ-K] Kit, https://www.cellsignal.com/products/proteomic-analysis-products/succinyl-lysine-motif-succ-k-kit/13764?N=102285+4294956287&Nrpp=30&No=30&fromPage=plp
3)PTMScan Malonyl-Lysine [Mal-K] Kit, https://www.cellsignal.com/products/proteomic-analysis-products/malonyl-lysine-mal-k-kit/93872?site-search-type=Products&N=4294956287&Ntt=malony+ptm&fromPage=plp&_requestid=710843

Eukaryotic cell lines

Policy information about cell lines and Sex and Gender in Research

Cell line source(s)	Mewo cells, JCRB Cell Bank, human malignant melanoma, male (78-year-old)<br>SEKI cells, JCRB Cell Bank, human malignant melanoma, female (28-year-old)
Authentication	None of the cell lines used in this study were authenticated in our laboratory.
Mycoplasma contamination	The cell lines were not tested for mycoplasma contamination in our laboratory.
Commonly misidentified lines (See ICLAC register)	No commonly misidentified cell lines were used in this study.

Animals and other research organisms

Policy information about studies involving animals; ARRIVE guidelines recommended for reporting animal research, and Sex and Gender in Research

Laboratory animals	Mouse: BALB/c nu/nu: BALB/cAJcl nu/nu, CLEA Japan, adult 8-week-old female and male mice for subcutaneous injections of MEWO or SEKI cells<br><br>Mouse: C57BL/6N: C57BL/6NJcl, CLEA Japan, adult 12-week-old female and male mice for starvation experiment<br>Mouse: C57BL/6N: C57BL/6NJcl, CLEA Japan, adult 12-week-old female for 5FU experiment, 6-week-old female mice for FK866 experiment<br><br>Mouse: male and female K-rasLSL/G12D/+:p53LoxP/LoxP (KP mice), produced by crossing K-rasLSL-G12D/+: B6.129S4-Krastm4Tyj/J mice (The Jackson Laboratory, Stock No: 008179; RRID: IMSR_JAX:008179) and p53LoxP: B6.129P2-Trp53tm1Brn/J mice (The Jackson Laboratory, Stock No: 008462; RRID: IMSR_JAX:008462), bred in the SPF breeding room of the Aichi Cancer Center Research Institute, genotyped at 4 or 5 weeks of age, adult 6-12-week-old mice for intratracheal infection of Adenovirus-Cre (Ad5CMVCre, VVC-U of Iowa-5, University of Iowa)<br><br>Mouse: male and female cis-Apc+/delta716/Smad4+/-, C57BL/6N background, PMID: 9506519, bred in the SPF breeding room of the Aichi Cancer Center Research Institute, genotyped at 4 or 5 weeks of age. Due to loss of heterozygosity, cis-Apc+/delta716/Smad4+/- mice spontaneously develop multiple invasive intestinal carcinomas and generally become moribund within four months of age.
Wild animals	This study did not involve wild animals.
Reporting on sex	Results of sex-based analyses were provided in Fig. S2 and S7.
Field-collected samples	No field-collected samples were used in this study.
Ethics oversight	All animal experiments were performed according to protocols (#27-9, #28-15, #30-3, #30-9, #31-14, and #R2-7) approved by the Animal Care and Use Committee of Aichi Cancer Center Research Institute.

Note that full information on the approval of the study protocol must also be provided in the manuscript.
